# A Case of Atrial Tachycardia Masquerading as Sinus Tachycardia in a Pregnant Female—A Case Report

**DOI:** 10.1155/cric/5523100

**Published:** 2024-11-14

**Authors:** Nismat Javed, Shoaib Ashraf, Ankita Gore, Mohammad Aziz, Muhammad Ali Aziz, Eduard Sklyar

**Affiliations:** ^1^Department of Internal Medicine, BronxCare Health System, Icahn School of Medicine at Mount Sinai, Bronx, New York, USA; ^2^Department of Cardiology, BronxCare Health System-Mount Sinai Morningside, Bronx, New York, USA

**Keywords:** atrial tachycardia, catheter ablation, management, supraventricular arrhythmia

## Abstract

This case report discusses the diagnosis and management of a 25-year-old pregnant patient presenting with persistent tachycardia. The patient, with a past medical history of thyroiditis, polycystic ovarian syndrome, and obesity, was admitted due to palpitations and was diagnosed with atrial tachycardia. Despite medical management with metoprolol, adenosine, digoxin, and flecainide, the tachycardia persisted, necessitating discussion about cardiac ablation. The report emphasizes that atrial tachycardia poses a significant clinical challenge when refractory to medical therapy. It also highlights the condition's association with tachycardia-induced cardiomyopathy and the role of catheter ablation in its management. This case underscores the need for a high index of suspicion for atrial tachycardia in pregnant patients presenting with persistent tachycardia and the importance of appropriate referral for invasive management when medical therapy fails. The case also highlights that atrial tachycardia in pregnancy can be safely managed with ablation.

## 1. Introduction

The prevalence of supraventricular tachycardia is estimated at 2.25/1000 persons globally [1]. Regarding atrial tachycardia, about 90,000 new cases are diagnosed annually in the United States [2]. Maternal SVT has an estimated frequency of 22/100,000 pregnancy-related hospitalizations in the United States [3]. Many mechanisms have been postulated for the development of atrial tachycardia, including increased automaticity, heightened sensitivity to *β* − 2 adrenergic receptors, the development of a microreentry pathway, and the expression of stretch-mediated channels [3]. Atrial tachycardia, whether sustained or paroxysmal, is initiated by an atrial premature complex [4]. The atrial rhythm is consistently rapid, but the physiological blocking of many ectopic p-waves in the atrioventricular node often leads to an irregular ventricular rate response [4]. Enhanced automaticity usually results in the precipitation of incessant atrial tachycardia that might lead to the development of tachycardia-induced cardiomyopathy [4, 5]. Atrial tachycardia, although benign, represents a challenge as the incessant forms can lead to cardiomyopathy and might require catheter ablation [5]. Overall, about 10%–15% of the patients referred for supraventricular tachycardias require ablation due to atrial tachycardias [6]. In this case report, we discuss the case of a 25-year-old pregnant patient who was admitted due to palpitations as a result of tachycardia and was subsequently diagnosed with atrial tachycardia refractory to medical management.

## 2. Case Presentation

**Figure 1:**
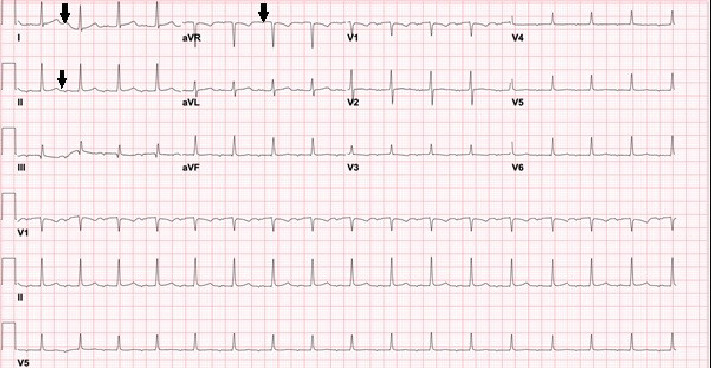
Sinus p-waves visualized on ECG (p-waves marked with black arrows).

A 25-year-old female with a past medical history of thyroiditis for which she completed thyroxine treatment, polycystic ovarian syndrome, and obesity presented to the outpatient obstetrics clinic for her prenatal check-up at 16 weeks of gestation. This was her first pregnancy. Her social history was significant for marijuana and alcohol use until a week before the presentation. She endorsed having palpitations associated with dizziness that worsened upon standing for the past 1 month. Her vitals on evaluation were within normal limits except for tachycardia (pulse of 123 bpm). Her physical examination revealed a gravid abdomen without any palpable contractions; the rest of the exam was unremarkable. Her initial labs on presentation were significant for mild anemia (11.3 g/dL; reference range: 12–16 g/dL) and elevated lactic acid (2.3 mmoles/L; reference range: 0.5–1.6 mmoles/L). The electrocardiogram on presentation is shown in Figure 1.

**Figure 2:**
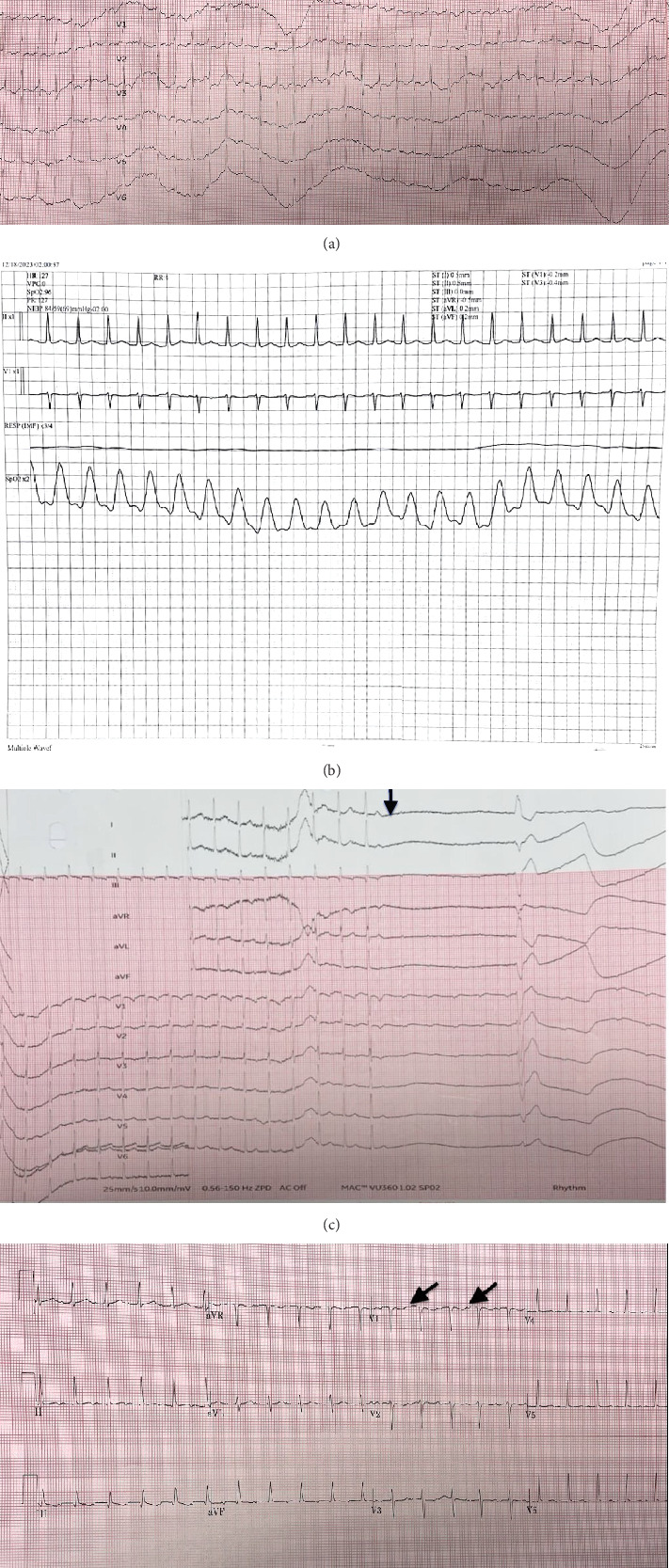
(a) Heart rate increasing before the polarity of p-waves changes. (b) Telemonitoring strip before atrial tachycardia. (c) Atrial tachycardia visualized by slowing of rhythm on adenosine administration (pointed by the arrow). (d) Nonsinus p-waves observed in V1 after adenosine administration (pointed by the arrows).

The echocardiogram on presentation revealed a mildly reduced ejection fraction of 46.5% (calculated using M mode) and indeterminate diastolic function owing to tachycardia ranging to 153 bpm. Additionally, the rate increased with activity, for example, when the patient tried to walk to the bathroom or during eating. Given these clinical features, differential diagnoses also included atrial tachycardia. The patient was admitted to telemetry for further monitoring. Metoprolol 25 mg every 8 h was started as a part of medical management, and adenosine was administered during telemonitoring, followed by ECG changes shown in Figures 2(a), 2(b), 2(c), and 2(d).

However, the patient's tachycardia persisted, and she was administered digoxin (500 mcg loading dose, followed by 250 mcg after 6 and 12 h of loading dose, respectively) on Day 2 of hospitalization. Subsequently, the patient was transferred to CCU for further monitoring. Flecainide 50 mg every 12 h was also started on Day 3 of hospitalization. Despite titrating flecainide and metoprolol, rate control was not achieved, and heart rate varied between 100 and 110 bpm at rest but increased to 190 bpm with mild activity. Flecainide was discontinued due to insufficient rate control as heart rate remained unchanged and concerns about potential side effects such as cholestasis of pregnancy or decreased fetal heartbeat variability. Despite the uptitration of medications as above and additional therapies, the patient's heart rate did not change, and she had more frequent episodes of atrial tachycardia. A multidisciplinary approach was advised. Aspirin was administered for the primary prevention of preeclampsia. She was transferred to another center for further monitoring and evaluation. Her EP diagnosis was focal atrial tachycardia stemming from the lateral wall of the right atrium. She underwent ablation during her pregnancy. Her follow-up ejection fraction at follow-up was 57%, leading to the reversal of cardiomyopathy.

## 3. Discussion

Atrial tachycardia in pregnancy has been documented in the literature; however, it has mostly been presented as a benign feature [3]. The age of presentation varied from 23 to 48 years of age in pregnant patients, similar to our patient [7, 8]. In one study by Hubbard et al., the fetus's gestational age was 15 weeks, with a varying spectrum ranging from 11 to 40 weeks of gestation, otherwise [9–11]. Most patients were either asymptomatic on presentation or presented with mild palpitations [3]. A previous history of supraventricular tachyarrhythmia predisposed patients to the development of atrial tachycardia in pregnancy, as discussed in five cases [12–16].

The electrocardiographic features in the literature vary. The rate of atrial tachycardia has been documented to be higher than 150 bpm [3]. In three cases, the rates were less than 150 bpm [10, 17, 18]. Common findings included the presence of nonsinus p-waves [3]. However, the features depended on the tachycardia's origin [3]. Common sites for the origin included right-sided crista terminalis and right-sided appendage [3]. Echocardiography also reported tachycardia-induced cardiomyopathy in 67% of the cases with incessant atrial tachycardia and subsequent improvement on intervention [3]. Sympathovagal discharges usually precede paroxysmal atrial tachycardia compared to sympathetic activation observed for sinus tachycardia [19]. The distinctive autonomic nerve activity patterns linked to various arrhythmias lead to the reasonable conclusion that ANA triggers paroxysmal atrial tachycardia [19]. Therefore, ECG tracings of atrial tachycardia are not expected to be interpreted as sinus tachycardia due to subtle changes in the polarity of p-waves. However, it is essential to note the difference in pathophysiology and, therefore, the difference in ECG changes.

During pregnancy, there are no explicit guidelines for using antiarrhythmic drugs. They are generally not recommended except in severely symptomatic cases. Limited randomized trials and systematic data exist on their efficacy and safety during pregnancy [20]. Considerations include potential side effects (such as QTc prolongation, exacerbation of tachyarrhythmias, and hypotension) and differential effects on both the mother and fetus [20]. Studies have shown fetal complications in gestational arrhythmias treated with antiarrhythmic drugs, including respiratory distress syndrome, growth restriction, and congenital heart disease [20].

Additionally, a retrospective study found a higher risk of placental abruption in pregnant women with arrhythmias [20]. Due to sodium channel blockade, flecainide, a Class 1C agent, has mild QT prolongation effects. Prolonged use of flecainide is linked to cholestasis of pregnancy and reduced fetal heart rate variability [21].

Medication failure has been commonly reported. Atrial overdrive pacing was also studied in some cases, and it failed to revert the tachycardia [9, 11]. Digoxin, verapamil, flecainide, metoprolol, propafenone, diltiazem, and procainamide were studied in most cases and failed when utilized for management [3]. In this regard, propranolol and quinine were effective in two cases [12, 22].

During pregnancy, focal atrial tachycardias primarily result from abnormal automaticity, typically occurring around 24–25 weeks of gestation [23]. However, recent findings reveal ivabradine sensitivity in a subset of atrial tachycardias with automaticity, with or without cardiomyopathy [23]. Though well-controlled studies in pregnant women are lacking, data from trials and postmarketing surveillance suggest a relatively low risk. Clinical trials in heart failure and coronary artery disease did not report fetal abnormalities, but there were cases of growth restriction and premature birth [23].

Effective management of incessant atrial tachycardia depends on further interventions, specifically catheter ablation [3]. Regardless of the focus of the arrhythmia, ablation using mapping had an overall positive outcome with minimal rates of recurrence and an overall improved prognosis [3]. In one case, excision of the right atrial appendage was necessary despite catheter ablation [3]. Complications depend on the position of the approach for the ablation; for example, there may be atrioventricular block and damage to the conducting system in strategies targeting the right side of the septum [3]. Three-dimensional mapping, however, provides a promising solution to these issues by reducing the amount of radiation administered and, therefore, reducing the amount of damage inflicted [24]. Our case discusses ablation as a safe management option in pregnant patients with atrial tachycardia when medications might pose a risk to the fetus.

## 4. Conclusion

Though atrial tachycardia is generally harmless and infrequent, it poses considerable clinical difficulties due to its unresponsiveness to medicinal treatment. It is often linked with tachycardia-induced cardiomyopathy. The successful handling of atrial tachycardia usually necessitates further interventions, particularly catheter ablation, when refractory to medical management. Despite the possibility of complications, ablation with mapping usually results in favorable outcomes with a low recurrence rate and an overall enhanced prognosis.

## Data Availability

Data can be made available on special request addressed to the corresponding author.
